# Use of Digital Tools, Social Isolation, and Lockdown in People 80 Years and Older Living at Home

**DOI:** 10.3390/ijerph19052908

**Published:** 2022-03-02

**Authors:** Adèle Gauthier, Cécile Lagarde, France Mourey, Patrick Manckoundia

**Affiliations:** 1 “Pôle Personnes Âgées”, Geriatrics Internal Medicine Department, Hospital of Champmaillot, University Hospital, 21079 Dijon, France; adele.gauthier@live.fr; 2 “Gérontopôle Pierre Pfitzenmeyer”, Hospital of Champmaillot, University Hospital, 21079 Dijon, France; ce.lagarde@orange.fr (C.L.); france.mourey@u-bourgogne.fr (F.M.); 3 INSERM U-1093, Cognition, Action and Sensorimotor Plasticity, University of Burgundy Franche-Comté, 21000 Dijon, France

**Keywords:** digital tool, feeling of loneliness, lockdown, older adult, social isolation

## Abstract

The COVID-19 crisis and associated lockdowns have exposed the extent of social isolation among older adults (OAs). Currently, the French government and medical, social, and charitable organizations are working to find means of limiting the multiple psychological and physical consequences of social isolation on the health of OAs. One proposal is to help the elderly become more comfortable using digital tools (DTs). However, the ability of DTs to reduce social isolation is disputed in the literature. This study aimed to collect the views of OAs on social isolation; to identify the determinants of the use or not of DTs, in particular in the context of a lockdown; and the role of DTs in the strategy to reduce social isolation. This qualitative study was based on 27 semi-structured individual interviews with OAs ≥ 80 years, in Côte-d’Or and Haute-Marne (French departments), from March to May 2021. A total of 96.3% of participants had already owned one or more DTs (mobile phone, tablet, or computer) for several years. The lockdown had not prompted the population to equip themselves more. The most common reason for using DTs was to maintain contact with relatives, and 63% of the participants said that DTs have a positive impact in reducing social isolation. However, there is a significant need for assistance and training in their use, especially since many services are now offered online. The participants suggested that the key to minimizing social isolation remained the maintenance of social contacts. In conclusion, DTs appear to be useful for helping the elderly maintain social links with relatives and, therefore, have a strategic place in the reduction of social isolation. However, these tools should not replace in-person interactions.

## 1. Introduction

In France in 2017, 900,000 people that were ≥60 years were experiencing social isolation; one third of these individuals were considered to be totally socially isolated because they had virtually no in-person contact with their main social circles [1]. In the world, about half of older adults (OAs) are at risk of social isolation [2]. The age of 85 can be considered the breaking point at which there is a drastic decrease in outings and physical contact [1]. However, the French population aged ≥65 continues to increase, as does life expectancy [3,4]. According to the Institut National de la Statistique et des Études Économiques, the population aged ≥75 will double between 2013 and 2040, and meanwhile the population aged ≥85 will quadruple [4]. Beyond life expectancy, disability-free life expectancy, which is more relevant to quality of life, is also increasing. In 2020, a 65-year-old woman could expect to live 12.1 years without disability and a man 10.6 years, which is an increase of 25 months for women and 23 months for men since 2008 [5].

Social isolation is defined as an objective lack of interactions with others or the wider community, while loneliness is defined as the subjective feeling of the absence of a social network or a companion [6].

Social isolation and loneliness have multiple consequences on health, both physical and psychological, and are significantly linked to an increase in all-cause morbidity and mortality, especially in OAs that are aged ≥65, comparable to smoking, obesity, lack of exercise, and arterial hypertension [7,8,9]. Social isolation and loneliness impact human well-being and quality of life [10], which is why there is a need to institute preventive measures to reduce isolation and the consequences of isolation in OAs.

The coronavirus disease 2019 (COVID-19) pandemic and the associated restrictions and lockdowns have exposed the extent of social isolation and its consequences, especially in the frailest populations, and illustrated the importance of social connections [11]. A report from the “Petits Frères des Pauvres” in June 2020 showed that 4% of OAs had no physical contact with their family during the first lockdown [12].

As part of a mission to identify the tools that are currently available to the public authorities to combat the isolation of OAs, a research firm produced a document with 36 proposals relating to several main areas, including listening to OAs, starting with their wishes and expectations, and promoting digital adaptation [13,14].

The use of digital tools (DTs) is heterogeneous in various population groups, even if the gap is starting to close [15]. It is legitimate to wonder if DTs are currently an efficient means of reducing isolation and loneliness of OAs.

Our study thus aimed to collect the views of OAs on social isolation and the role of new technologies, including information and communication technologies, particularly in the context of the lockdown. This work also sought to identify the determinants for whether or not DTs should be used in the strategy for reducing social isolation in the elderly. To carry out this study, we used the theoretical framework for social isolation, defined as an objective lack of interactions with others or the wider community, and loneliness, defined as the subjective feeling of the absence of a social network or a companion [6].

## 2. Materials and Methods

### 2.1. Study Design

This qualitative, descriptive, multicenter study was conducted between 11 March and 15 May 2021, in the form of semi-structured individual interviews.

The items of these interviews were developed from the literature that was reviewed. These interviews made it possible to collect the necessary data, including socio-demographic data; the use of DTs; the description of the relational network with the perception of its quality, i.e., the existence of social isolation or feeling of loneliness; and its evolution during the COVID-19 pandemic.

### 2.2. Population

The population consisted of adults ≥80 years, living at home, in the departments of Côte-d’Or and Haute-Marne (France) and recruited through the associations France Alzheimer Côte-d’Or and Familles Rurales 52, or through one of three general practitioners (GPs) with a private practice in the Dijon urban area.

The only non-inclusion criterion was the existence of a major neurocognitive disorder.

The people that were interviewed were progressively included in the study until a data saturation effect was reached, i.e., when the analysis of the collected data no longer provided new elements to the current research, thus defining the total number of participants. Bloor & Wood have defined the theoretical saturation as the continuation of sampling and data collection until no new conceptual insights are generated. At this point the researcher has provided repeated evidence for his or her conceptual categories [16]. This study was conducted in accordance with the Declaration of Helsinki and French national standards. The Ethics Committee of our institution was consulted and approved this study, which was outside the field of Jardé’s law. Consent was obtained from all the participants, and the data were processed anonymously.

### 2.3. Interviews and Collected Data

We collected data relating to family status (including marital status), social environment, leisure activities, and profession before retirement. The participants were then asked about whether they owned and used DTs, including mobile phones, tablets, and computers. The term DT was explained to participants by listing the three devices. The survey was based on the interview guide that was developed with one of the authors who has a PhD in Sociology (Table 1).

To assess the participants’ social network, we used an objective indicator, the social density score (SDS) of the Centre de Recherche pour l’Étude et l’Observation des Conditions de vie (CREDOC), which results in scores varying from 0 to 8 (Table 2) [17].

All the interviews were performed by the same investigator and recorded with a digital voice recorder, then transcribed in full. Non-verbal elements (silences, onomatopoeia, emotions) were also noted. The verbatims were transcribed literally, with no correction or reformulation.

### 2.4. Data Analysis

The quantitative analysis was done manually using a double-entry Excel^®^ spreadsheet (Microsoft Corporation, Redmond, WA, USA).

A first qualitative analysis was performed through two readings of the verbatims. The first reading, that was done by the interviewer, consisted of a verbatim transcription of the interviews. Then, a second reading was done by a specialist in sociology to improve the validity of the results.

The second qualitative analysis was done using Sonal^®^ software (Xavier Le Nué, Tours, France), which allows the interviews to be encoded. It consisted of dividing the participants’ responses into several themes corresponding to the interview guide and then producing a summary by theme.

## 3. Results

The main results are summarized in Table 3.

### 3.1. Population

The interviews, which lasted between 18 and 56 min, were carried out until a data saturation effect was reached. This effect was obtained after 27 interviews.

A total of 17 participants (63%) were women. The mean age was 85.9 ± 5.3 years (80 to 99).

A total of 15 participants (55.6%) lived in rural areas.

A total of 15 participants (55.6%) lived alone (10 widows, four divorced, and one single) and 12 (44.4%) lived as couples.

All of the participants were retired. A total of 22.2% had exercised a middle management, 18.5% were executives, 14.8% farmers, 14.8% employees, 14.8% blue-collar workers, 11.1% artisans/merchants/business managers, and one was a housewife.

A total of 17 participants (63%) reported a weekly or bi-monthly visit from a housekeeper, two had help with personal hygiene, and one received home meal deliveries. A total of 10 participants (37%) did not have any help at home.

A total of 24 participants (88.9%) had visual disturbances and wore glasses and 14 (51.8%) reported hearing impairment and wore a hearing aid.

A total of 19 participants (70.4%) went out of their homes daily and walked regularly, with or without technical assistance. Of these, 16 (59.3%) were still driving. A total of eight individuals (29.6%) had difficulty walking and rarely left their home, including one who nevertheless regularly used her car.

### 3.2. Environment before Lockdown

Of the 27 participants, 14 (51.8%) saw their family members at least once a week, six (22.2%) once or twice a month, and seven (25.9%) less often.

A total of 18 participants (66.6%) met up with their friends at least once a week, and nine (33.3%) only once or twice a month.

A total of 14 participants (51.8%) belonged to several associations or sports clubs and 9 (33.3%) belonged to one association. Participating in an association was perceived as essential for 24 participants (88.8%), punctuating their week, and maintaining social links. For instance, Participant 27 (P27) said: “Thanks to France Alzheimer and its support groups, I never feel alone”.

The mean SDS was 4.5 ± 1.6/8. A total of four participants had an SDS of 7, five an SDS of 6, four an SDS of 5, five an SDS of 4, six an SDS of 3, and three an SDS of 2.

### 3.3. Feeling of Loneliness before Lockdown

To the question “Before the lockdown, did you experience a feeling of loneliness?”, 24 participants (88.8%) answered negatively and three positively. The three positive responses were all women, aged 81, 84, and 87, including two widows living alone and one living as a couple with geographically distant children. Before retirement, they were a headmaster, housewife, or laboratory technician. Their SDS were low at 2, 3, and 5. The 24 OAs who answered negatively declared being active on a daily basis and trying to develop strategies to minimize loneliness. P2 said, “A day without seeing anyone for me is deadly. I always manage to go out or go shopping. I tried to find solutions”. P5 said with a laugh, “I have too much to do, I don’t have time to feel alone”. Finally, P21 confided “We lived on a high note” and added “we went out so much every day that… no. Socialization is so important, you have to go out”. Nonetheless, four of these 24 OAs reported having a fairly solitary temperament and felt that loneliness was not negative, such as P16 saying “I recharge my batteries a lot by being alone”.

Leisure activities such as reading, painting, or board games were part of the daily life of all the participants. Social connections (physical meetings or through DTs) with their families or friends was, according to all participants, the best remedy against loneliness.

For five people, feeling lonely was also a matter of character and willpower, even going so far as to be seen as a weakness. P26 said “I think it’s a matter of genes. We don’t mourn in the family, we don’t complain”. P14 added “I don’t mope, it’s not in my personality”. Always P14, who associated feelings of loneliness with depression, said “Me, nah, I’m in good spirits; I’m not depressed”.

### 3.4. DTs Owned

#### 3.4.1. Equipment and Connections

A total of 26 of the 27 participants were familiar with the three DTs (mobile phones, tablets, and computers).

The mean number of DTs per person was 2 ± 0.8.

A total of 26 of the 27 participants had a mobile phone, and 15 participants had smartphones. Of the 12 participants with a tablet, two reported minimal use. Of the 14 participants with a computer, two reported minimal use. A total of 10 had all three DTs, 9 had two, and 7 had only one. Only one person had no DTs, only a landline.

A total of 17 participants had an internet connection. No participant ≥ 90 years had an internet connection.

#### 3.4.2. Acquisition and Learning to Use DTs

Of the 26 people that were equipped with DTs, 25 had acquired them several years ago, dating back to the 1990s, and only one had acquired their tablet during lockdown in 2020. A total of nine people (33.3%) had learned to use them in the workplace, a few months or years before their retirement.

For 65% of the participants, DTs were acquired voluntarily, in order to “stay in the game”, as stated by P15. For the remaining 35%, the DTs had been given as gifts.

Safety, especially when traveling by car, was the most common reason for purchasing their first mobile phone. A total of seven participants (25.9%), especially those aged ≥90, citied this reason. P11 explained “When I go down into the basement I always take it [mobile phone] with me in case something happens to me”.

Half of the 26 equipped participants were taught to use DTs by their descendants. A total of three OAs found that the explanations that were provided by family members were not very helpful because it went too fast and was not pedagogical. P18 said “My son explained a little to me, but he gave me a lot of information and he doesn’t have a lot of patience my son, so it annoyed him because it didn’t stick”. P13 said “My children don’t realize, we never worked with that”. P26 said “Now I’m going back to school! When my granddaughter explains to me it’s okay but as soon as she leaves I can’t do it anymore”. The other half said they learned “on the job”. P15 said “We are trying to get by, by fiddling around”. A few had developed strategies to improve their skills in DTs, notably through books on their vocabulary and usage. A total of eight OAs (29.6%) had participated or were still participating in digital workshops.

### 3.5. Use of DTs

#### 3.5.1. Functions Used

A total of 21 of the 26 users (80.7%) of DTs described daily use. P13 said “It’s my life companion, it’s an indispensable tool, it’s one of the things I put in my suitcase when I go somewhere”. A total of five used them weekly to communicate with relatives or for ad hoc tasks such as viewing photographs or printing.

The 26 users mainly used DTs for communication (calls, messages, videoconferencing). A total of 15 participants used social networks, mainly Facebook^®^, WhatsApp^®^, and Skype^®^. Software for searching for information on the internet (recipes, DIY, facts or anecdotes on history, celebrity biographies) came in second, perceived as a real “source of knowledge”, as described by P2. For instance, P15 declared: “The other day I looked on the internet to clean my siphon, I’m a real handyman”.

Games (cards, crosswords) were also used. Word processing software and photo management and retouching programs were used incidentally. Online administrative procedures (bank, insurance, tax declaration) were used by 13 participants (40%). Finally, two participants made purchases on the internet.

#### 3.5.2. Objectives

Communication with family, friends, and acquaintances was the most common reason for using DTs (92.6% of users), highlighting the importance of maintaining social connections. 92.3% of the users knew how to make calls with the mobile phone and 76.9% could send text messages. Regarding videoconference calls, mainly discovered during the lockdown, P13 said: “It’s not like a phone call because you see them, it’s like they are there”. P15 confided “I find it great to have them, what joy! Can you believe it? My English grandchildren who I haven’t seen for a year, well I see them on video, it’s amazing”. P1 said “I think they bring life anyway, I was very happy to have them when I was in the hospital. With my family, we found this link to keep in touch”. P10, who owns of a mobile phone without internet access, wanted to equip herself with a tablet because “My nephew sees his son in America with his children. When they come to see me, I think they’ll try to do something about it for me. There’s just been a little Nina, she was born eight days ago and then there were twins in America, then little Peter. But as long as I know how to do it, that’s it; I just have to grasp it”.

A total of 18 users (69.2%) said they used the internet to search for information for leisure, in order to improve their general knowledge and skills. Taking and viewing photos on tablets and listening to music were activities that were enjoyed by 16 users (61.5%). A total of four users (15.3%), who were passionate about literature or retouching photographs, used specialized programs. P18 confided “There has been a shift from film photography to digital, it is extraordinarily practical”.

A total of five people said that the different functions used (social networks, games, information search) helped maintain cognitive function. P26 said “It makes your mind work; it’s good for memory”. P18 said “Having conversations keeps the brain alive”. P3 confided “It made my brain work”.

By using DTs, 19 participants (70.4%) had a real desire to stay up-to-date/connected to the world and to follow the current technological trends. According to P15: “This is the future. I mustn’t die stupid. I must learn. I had to know something better. For example, my sister who is 13 years younger than me doesn’t care. I wanted to learn”. P2 said “It’s the evolution of society, all that”.

#### 3.5.3. Reasons for Not Using DTs

For 10 participants who did not have internet access and those who did not use DTs or only rarely, the reasons for not using them were:

- Lack of interest (expressed by eight people). P5 confessed “It’s not for me. As long as I can drive I don’t need anything else”, while P27 clearly displayed his opposition, saying “It is a choice, I don’t want to be addicted … I want to keep my freedom”.
- The lack of family or the family not encouraging the use of DTs (for five non-users). P5 said, “It didn’t interest me. But, maybe if I had children or grandchildren it might have been”.
- Fear of not being able to use it, especially among those that were aged >90. P10 said “Yeah, I would like a tablet but I don’t know if I could use it”.
- Fear of scams. The participants were very apprehensive about the risk of internet scams, citing stories from the media or mishaps that had happened to their relatives. They saw OAs as easy victims and that a certain level of skill was needed to avoid being scammed. P5 declared “People get taken in with this … the scams all that. I say you have to be young. I don’t know if I would understand anything in all of this”.

### 3.6. Participants’ Views on DTs and OAs

#### 3.6.1. Use of DTs by OAs

A total of 24 participants, including non-users, saw DTs as positive and beneficial as long as the individual knows how to use them. P13 stated “I say that it is a chance for those like me who were trained, we are privileged. But, I think it is a pity for the others. Because, I know a lot of people who do not use them, who are afraid”.

The term DT took on its full meaning since the devices were often described as a useful “tool”. P9 “It’s wonderful. I have friends who are the same age as me and who are against all that. It’s a real tool, like a hammer [laughs]. It seems essential to me, even at our age. And it keeps you busy”.

The possibility of being able to maintain a social link and interact with relatives was essential, even if most participants preferred to see their relatives face–face. P4 said “It’s very good but it’s still virtual. I would rather have my grandson in front of me, but hey”. New technologies were seen as an additional way to communicate with their family, though not replacing real meetings. According to P22: “Once we have tasted it we will not do without. It allows an exchange anyway. My neighbor sends me messages. It makes a contact. But, well, the best is still to go see people”. P26 said, “It’s great, otherwise we would be isolated. If you saw the number of people I chat with on the internet. It is a great service, especially at the relational level.” P17 spoke of the current phenomenon in which families are spread out, saying “At the time, families were not as broken up as they are now, everyone lived under the same roof”.

The older participants clearly verbalized the importance of adapting to societal changes. So, to the question “What do you think about the use of DTs by OAs?”. P27 replied “Like all new things, it brings something, it meets a current need”.

#### 3.6.2. Risks of Use

The rather favorable opinion of the use of DTs was balanced by certain risks. For instance, P11 addressed the risk that was associated with overuse, “It’s rather something good from the moment you are measured in how you manage your day. But knowing that people lock themselves in an office for five hours does not seem very positive to me”.

The excessive use of digital technologies was described as dangerous, such as P27 who said “It colonizes you very quickly, you quickly become dependent on it”. Some even used stronger terms, like P5, whose idea was not to misuse DTs because “We would become slaves to them”.

The excessive time that is spent on screens, especially during times of the day when the family gets together and is supposed to talk, such as meals, was considered deleterious to family life. Several participants said they were afraid that these virtual contacts would take precedence over the real exchanges of everyday life, as expressed by P17: “In the century of communication, we no longer communicate”.

Among the couples that were interviewed, the use of DTs was sometimes a bone of contention, in particular when one of them used little or no NT, such as the spouse of P20, who confided “He spends so much time on the computer that we no longer communicate, sometimes he doesn’t even hear me when I speak to him. It taints our life as a couple”.

A total of 48.1% of the participants mentioned the dangers of social network. Some cited the issue of violence between young people that is reported in the media. P11 said “When we see these exchanges between these young people who attack each other, it is very dangerous”. This was corroborated by P22: “We see everything and anything. Everyone has access to it so it goes very quickly”.

For 22.2% of the participants, access to personal data provided in real time by these websites was considered intrusive. P21 explained “It bothers me a bit because everyone posts what they do and everyone has access to that. I find that it lacks privacy” and added while joking “With all the networks now people are monitored. Personally, I wouldn’t like my children to know where I am every moment”.

The use of social networks was seen by four people as an illusion of human contact, without real friendships. P9 described them as “anti-social networks”.

The two oldest participants, at 99 and 94, added that social networks were sometimes used for narcissistic, self-centered purposes. P27 suggested the following: “Look a little bit. Young people are more and more isolated, young girls show naked images of themselves on the internet. I think they‘re missing something, maybe a relationship with their parents, and that’s why they look all over the world for connections. People are looking for support everywhere”. P19 agreed: “I think people risk isolating themselves more with this. They are typing on it [the internet] all the time, looking for information, and then they think more about social contact. In my opinion, it is a personal contribution; it’s not a social contribution”.

A total of two people raised the subject of false information provided by advertisements on the internet or on social networks, which could be dangerous for beginners and unsuspecting users. The term “fake news” was also used by two individuals, including P9, who declared that he was “tired of being constantly on the lookout for lies”.

P18 shared his desire to improve the reliability and security of DTs: “I think NT, smartphones should be made more secure, for young and old”.

One person spoke of the danger of the electromagnetic waves that were emitted by devices.

P22 concluded with a particularly interesting expression, “DTs are like all tools, if you use them badly you can get hurt”.

#### 3.6.3. OAs and Support for Using Digital Technology

A total of nine participants (33.3%) said they no longer did administrative tasks and let their children take care of them. Of the 18 who still completed their own tax returns, five did so on paper and 13 did so online. A total of nine participants required the help of relatives and felt lucky to have this help.

To the question “What do you think of the support provided to OAs in our society, which is becoming more and more digital?”, the response was almost unanimous, with 92.6% of participants answering that OAs did not have enough help to be able to perform administrative tasks online.

The digital transition was often found to be difficult, as for P2 who said “It can cause big problems; it is a source of enormous distress in the face of these new things that are imposed on us”. P26 cited the example of making an appointment to be vaccinated against COVID-19: “For vaccines, my son made an appointment for me on the internet because I couldn’t get an appointment by phone. I could never have been vaccinated otherwise”.

#### 3.6.4. Digital Workshops

A total of eight OAs (29.6%) had already participated or were participating in digital training, including six in weekly group workshops, and three individually with a teacher at home.

A total of four of the six participants in the group workshops had a mixed opinion, such as P3, who was unhappy that the training was not done on her own material: “First, it didn’t teach me much, and then they lent us a computer over there and when I went home it wasn’t the same”.

The three people who participated in personalized training were all very satisfied. This was confirmed by P26: “But there is a young teacher who comes to the house, that’s very good. It gives us confidence, because we don’t dare to do things”.

A total of two OAs did not wish to participate in a training course because they thought they were too old.

### 3.7. Lockdown Experience

A total of 16 of the participants (59.3%) declared that they were not impacted by the lockdowns or very little. Those who lived as a couple considered themselves lucky; living together helped them to better manage the social isolation resulting from the lockdown. The people who were less mobile overall told us that they had not noticed any changes. A total of two people equated this period with the restrictions that were suffered during World War II. P5 explained “Oh you know, we old folks saw others during the war” and P13: “It didn’t scare me because I remember the war”.

A total of 11 participants (40.7%) reported having suffered from the cessation of associative activities and from the distance from their family. P27 mentioned the consequences of the feeling of loneliness on his cognition by saying “You only have television-type contacts, so you lose your mind. I am missing syllables when I speak; my vocabulary is restricted. It is extremely painful to live with. If I didn’t have some very close friends here I would have lost it”.

A total of 18 OAs (66.7%) developed strategies to combat boredom and isolation, by finding different activities to fill the day. On the relational level, being for the most part having been equipped with DTs for several years, they used them to maintain social connections. A total of 11 OAs (40.7%) declared that the DTs had been essential for them during the lockdown. P13 said “It helped me decrease my loneliness. It allowed me to communicate, I’m lucky”.

### 3.8. Role of DTs in Reducing Social Isolation of OAs

A total of 17 participants (63%) said that DTs had a role in reducing the social isolation of OAs, and eight (29.6%) thought that they did not.

The communication tools that are offered by new technologies were seen as additional means to maintain social links by 63% of participants, as confirmed by P11: “Even if it is by telephone, a relationship is important”, and P18 declared “Look, for me, every Wednesday it’s Skype. We reduce isolation with this”. P12 extended this idea to OAs living in nursing homes: “I tell myself that in nursing homes, if older adults could have that, it would be good. For me, for example, if one day I move to a nursing home and, well, I’ll go with my phone and my tablet. It will be essential, I couldn’t do without”.

A total of 11 participants (64.7%) nonetheless touched on both sides of the argument, agreeing with the view of the eight people who answered negatively. For them, DTs are useful but had their limitations and were not always sufficient to reduce isolation. P18 said: “In part, but it’s not enough. There is something indispensable in humans, it’s direct contact. It is inscribed in Humanity”. P2 said “It’s a tool, it’s like a crutch. And my crutch didn’t give me back my leg, but it helps me walk”. According to them, the essential remedy against isolation was to maintain the social fabric by means of in-person meetings, with relatives or gathering together through association or community-based initiatives. P3 said, “A lot of people suffer from not seeing other people, that’s what is important”. P5 argued, “You have to participate in clubs—it’s getting together with other people that’s good. It helped me a lot”.

Finally, five people insisted on the need for OAs to keep reaching out to others.

## 4. Discussion

The use of DTs and their role in the strategy to reduce social isolation in the elderly are current subjects that are of interest to the medical, social, and political worlds; hence the interest of our study. The lockdowns during the COVID-19 pandemic have brought the issue of social isolation to center stage, prompting public authorities to consider numerous action plans. While studies have looked at the impact of using DTs or social networking, they included much younger people and/or special populations and/or targeted a smaller field [18,19]. Others were performed before the lockdown and with relatively younger populations [20,21,22]. Thus, to our knowledge, only two French studies from the same organization have analyzed at the discourse of OAs ≥ 60 years on their living conditions and the role of DTs during lockdown [12,23]. The prospective nature of our study had the advantage of circumventing potential issues with memory for the collection of information. Recruitment from two sources (members of charitable associations and patient base of GPs) improved the representativeness of the sample. The multicenter nature is another strength of the study since we were able to enroll participants from both rural and urban areas.

Our study population was very old (mean age 86). It was comprised of 62.9% of women, which is in accordance with French demography as of 1 January 2021 [3]. The distribution between the rural and urban areas was almost balanced, slightly in favor of the rural areas (55.5%). Finally, the distribution between the different socio-professional categories before retirement was fairly homogeneous.

In a normal context (no lockdown), the participants maintained a significant social network in the various circles of sociability (meeting mostly weekly with their family and friends). Except for the charitable associations, in which our survey population was more invested (85.1% vs. 55%), our results are similar to those that were reported by the study from the “Petits Frères des Pauvres”. They showed that in 2017, in a population aged ≥60, 22% were isolated (i.e., only a few physical meetings per year) from family, 28% from friends, and 21% from neighbors [1].

In our study, the mean SDS was 4.5, while it is 4.7 in the general population and 3.5 in people that were aged ≥70 according to a report from the CREDOC [17]. Thus, the sociability of our study participants was more similar to that of the general population than to that of individuals aged ≥70. This could be explained by the profile of the participants in our study, who were devoid of major neurocognitive disorders and mainly recruited in associations. They mentioned the importance of maintaining an active social life, whose physical or virtual preservation was essential, despite their advanced age. The only three participants who reported having feelings of loneliness scored SDSs of 5, 3, and 2, relatively low scores for the last two. In addition, these individuals had little or no family nearby. The feeling of loneliness, therefore, appeared to be quite correlated with the objective density of the social network. However, feelings of loneliness did not appear to increase with age, since these participants were 84, 87, and 81 years old. One study in the general population aged ≥15 reported that feelings of loneliness and boredom were more prevalent in isolated individuals compared to non-isolated individuals (18.1% vs. 7.7% and 17.1% vs. 7.6%, respectively). In the same study, despite a trend, the difference was not significant in individuals that were aged ≥70 [24].

In our study, the feeling of loneliness did not seem to be associated with the intellectual character of the profession that was exercised before retirement. A study showed that compared to those who were not enrolled, OAs studying in the “university of the Third Age” program for retired adults had a lower tolerance for uncertainty, with greater needs in conversation and social interaction and a lower capacity for decision-making in ambiguous situations regarding social interaction [25]. The needs in conversation and social interaction, including for decision-making in ambiguous situations, could be the expression of a feeling of loneliness.

A total of 40.7% of the participants declared having suffered from the lockdowns, which is lower than the 81% who claimed that the lockdowns negatively impacted their mental health in a study by Daly et al. [26]. The presence of a spouse was very beneficial. Most participants had maintained their usual indoor strategies against boredom (gardening, reading, sewing). The participants appreciated DTs more during the lockdown because they could see relatives by videoconference. Technology has been shown to help alleviate loneliness in OAs without direct interaction with other people, especially in the context of COVID-19 which has resulted in various social restrictions [27]. For 11 participants, stopping all outdoor activity caused major psychological suffering; digital technology failed to compensate for the lack of human contact. The negative impact of lockdown was felt in the physical, psychological, and social dimensions [11].

### 4.1. DTs Owned

The participants were well equipped with DTs since only one did not own any of the three DTs that were mentioned in our survey. The most common reason for purchasing DTs was to be able to call for help, especially in individuals ≥90 years, who were more fearful when going out.

Most participants had adapted to the progression of digital tools and communication; 62.9% of the population had an internet connection, 10 people had all three devices and 9 had two.

Among the 10 participants who did not have an internet connection, half lived in rural areas and the other half in urban areas, so the area of residence did not seem to affect this choice. In contrast, people who worked in low-income occupations had fewer digital devices. It is recognized that compared to people from lower socioeconomic strata, individuals from a higher socioeconomic level have a better access to digital technology and also more often possess the skills to used them [28].

None of the five participants ≥90 years had an internet connection. A total of three of them had only a mobile phone and one had a computer and a mobile phone. There appears to be a difference in the digital equipment of subjects that were aged 80–90 and those that were aged ≥90. It is legitimate to think that the older age group, who were around 60 years old when digital technology was introduced, did not have access to it while they were still working and did not take the opportunity then to equip themselves.

In our study, the lockdowns did not encourage participants to be better equipped.

Early adopters of DTs were also the most comfortable. Most had trained on their own and had kept up to date with digital developments. Similar to our study, Mitzner et al. found that OAs’ preferred method of learning to use the internet is self-learning, followed by help from family members [29]. For people who were equipped late by themselves or who were equipped by their relatives, getting started with DTs was more complicated, in particular due to lack of knowledge of the specialized vocabulary, as the literature reveals [30]. Several participants said that training with family had not been very effective due to a lack of pedagogy and patience. As reported in the literature [30], they described a generational gap; their children and grandchildren did not realize that it was challenging for them to understand the vocabulary and how to handle the devices. This feeling was also found in the study that was carried out by Portz and colleagues [31]. In the end, these people learned on their own. As shown in some articles, being taught to use the internet by a family member is not the preferred method for OAs [29].

According to the participants, the advantages of digital workshops in groups were the richness of the teaching and the social connections. However, they did not like using DTs other than theirs, the need to leave their home to participate, or the standardization of the lessons. The advantages of the individual workshops were the ability to do it at home, learning on their own material, and the personalized lessons. Nevertheless, six participants were unaware of the existence of digital workshops. A total of 11 individuals suggested that these workshops should be offered nationwide. This confirms the importance of training OAs to use DTs, which can improve their social inclusion and reduce feelings of loneliness, as reported in the literature [19,32]. Finally, strong arguments must be developed to encourage the oldest individuals to take part, since advanced age was mentioned as a barrier to learning.

### 4.2. Use of DTs, including during Lockdown

A total of 80.7% of our study participants used DTs on a daily basis, and the remaining 19.3% used them weekly. All the participants took them on vacation and to the hospital. The rate of DT use here was higher than the rate that was reported by the “Petits Frères des Pauvres” study. This study, which involved 1512 questionnaires from people that were aged ≥60 between April and May 2020, i.e., during lockdown, found that 75% of participants used the internet [12]. It also showed a decrease in internet use with increasing age, with 44% of users aged ≥80 and 33% aged ≥85 [12]. This is similar to our results, which found a difference in equipment and internet connection between OAs that were aged 80–90 and those aged ≥90.

Communication with family/friends/associations was the main reason (92.6%) for using DTs, as shown in the first “Petits Frères des Pauvres” study [12].

The main functions that were used by OAs were communication tools of all sorts (short message service, calls, video calls). In addition, 15 of the 27 participants used the main social networks. Videoconferencing calls were mainly discovered and used during lockdown, and the ability to view video images during a call with relatives was particularly appreciated. DTs had an important role in maintaining the social links of OAs, as reported in the literature [33], and increased their feeling of safety [34]. DTs were also used to look for information, to enrich general knowledge, or to improve daily life (cooking, DIY), this convenience of DTs being mentioned in the literature [35]. According to five participants, communicating or searching for information via DTs was important for preserving cognition. There are more and more tablet-based gaming platforms targeting OAs, aiming to improve their overall wellbeing by stimulating cognitive functions and promoting social interaction between players [36]. Finally, the population had a real desire to keep up with changes in society. This confirms the literature data: studies show that OAs with positive views of the utility of self-monitoring analysis and reporting technology are more likely to use it [37], particularly those that focus on health-related conditions. In addition, OAs were more likely to use applications if they could be extended to activities that were relevant to their everyday life (communication with others, information-gathering on topics of interest), and staying up-to-date with current events and social interests [38]. Peek and colleagues reported that the level of DT use by OAs is impacted by six main topics: challenges in the domain of independent living, behavioral options, personal thoughts on technology use, social network impact, influence of organizations, and physical environment role [39].

A total of ten participants did not have an internet connection and five used DTs infrequently because of an initial lack of interest. This lack of interest is also reported in the literature as the main reason for not using DTs [12]. In our study, older participants feared that they would be unable to use these tools due to their complexity, or because they were too old to make it worthwhile. Currently, many OAs are not familiar with computers and many remain skeptical about these new, virtual means of communication [11].

### 4.3. Participants’ Opinion of DTs and OAs

Most participants considered that DTs were beneficial to OAs, describing them as important tools, especially for communication, as reported in the literature [33]. All the participants, whether they were users or not, said they met a current need, including for OAs.

Overuse and dependence were the main issues that were raised by the participants, who sometimes used strong language such as “colonizing”, “slave”, or “dependent”. The participants described the younger generations as being addicted to NT, and, therefore, cut off from the real world and locked in the virtual world. The participants were worried that DTs would supplant intra-family communication. In couples, the risk of excessive and unbalanced use of DTs was also mentioned. Finally, everyone found DTs useful for maintaining contact with relatives, as long as they did not replace face-to-face meeting.

Most participants were familiar with social networks, especially Facebook and WhatsApp. Since they make it possible to maintain social links and emotional ties, the use of these networks to combat loneliness and isolation in OAs is recognized [11]. However, when participants were describing them, they brought up the ideas of dangerousness and intrusion because of the sharing of personal data. The relationships that are created through these virtual networks were considered futile, without any real connection. The violence on these platforms, especially between young people, was also mentioned, as well as several events with dramatic repercussions that were reported in the media. Moreover, the two oldest people felt that private information was shared for narcissistic purposes and in order to raise one’s own self-esteem through flattering comments from other members. Finally, the circulation of false information and fraudulent emails was another danger that justified not using them. According to the participants, they are ideal victims because they are untrained and less alert, so not sufficiently experienced to spot possible scams. This led to the suggestion that interfaces should be more secure to limit the risk of scams. These privacy and safety issues are also highlighted in the literature [40].

All the participants denounced a lack of adequate support in society for OAs by public services, faced with a rapid digital transition, generating anxiety, and a difficult experience of performing administrative tasks online. Programs such as the “university of the Third Age” could be one of the solutions. Indeed, it has been shown that such programs positively affect the life of seniors by stimulating them intellectually, socially, and physically, and trains them to cope with difficult situations, thus minimizing the danger of social exclusion [41].

### 4.4. Role of DTs in Reducing Social Isolation and Feelings of Loneliness in OAs, including during Lockdown

Several participants recalled finding the lockdown difficult and that it led to a vicious circle of gradual withdrawal from socializing. They added that, for them, loneliness could be the reason for admission to a nursing home. In the first “Petits Frères des Pauvres” study, 94% of participants considered that the fight against isolation is important for OAs [12].

According to 63% of participants, new technologies could help to reduce social isolation, as reported in the literature [42]. Indeed, DTs offer new means of communication for people living at home or in nursing homes. However, most said that virtual communication should not replace in-person meetings and participation in associations, as confirmed in the literature [11]. In the second “Petits Frères des Pauvres” study, although the usefulness of DTs to preserve audio and visual contact during lockdown was acknowledged, the participants considered them a temporary solution [23]. This was reinforced by the help initiatives (shopping, phone calls, moral support) that were introduced for seniors during lockdown. These initiatives were particularly appreciated by OAs because they fostered feelings of support/consideration, and reinforced feelings of belonging in society [23]. Furthermore, DTs alone cannot help an isolated OA without friends or close family to come out of isolation, because they have no one with whom to communicate. We deduce that digital communication is only useful in reducing social isolation if an OA is already anchored in a stable social circle. Another necessary condition for maintaining a good quality social network is the maintenance of the desire to be part of society. The first “Petits Frères des Pauvres” study showed that DTs are important vectors of social connections for two thirds of internet users [12]. In this latest study, the participants declared that the DTs, although useful for coping with isolation due to the lockdown, were not essential, and, for 87% of respondents, the absence of digital tools at home was not perceived as a problem [12].

A randomized controlled trial (205 OAs, mean age 82.8) showed that internet use was associated with lower feelings of loneliness in OAs that were living in senior residences, independently or with assistance. However, no statistical link existed between the use of digital tools and social isolation [43]. Another recent study (738 people ≥ 60 years) showed that those experiencing loneliness were more at risk of psychosocial distress and were less involved in community life. There was no statistical relationship between the use of information technology and psychosocial distress, feelings of loneliness, or belonging. In contrast, the use of this technology by the most isolated people was associated with greater psychosocial suffering [44]. A 2017 literature review did not find a sufficient statistical link between the use of DTs and the reduction of social isolation in OAs to establish reliable recommendations [45].

While they are clearly linked, social isolation and feelings of loneliness are distinct concepts. Social isolation refers to the feeling that one does not belong to society, while feeling of loneliness is the impression that one’s social network is poor or is smaller than desired [46]. In addition to their social effects, social isolation and feelings of loneliness influence physical and mental health, particularly in OAs [32]. While social isolation affects 6% of OAs ≥ 60 years in France, a feeling of loneliness is reported by 9% of individuals that are aged 60–74 and 17% of OAs ≥ 85 [1]. Feelings of loneliness and social isolation are associated with reduced physical performance [47], and a risk of loss of mobility and independence [32,48]. An insufficient social network is associated with a risk of heart disease and stroke through increased active smoking and physical inactivity [49]. In addition, social links might be associated with a reduction in blood pressure in healthy adults [50]. Social isolation is associated with a risk of cognitive decline in Oas ≥ 65 years that are living at home [51], and loneliness is recognized as a significant risk factor of depression in OAs [52]. The perceived quality of life is positively influenced in OAs by a strong social network and negatively influenced by loneliness [53,54]. Finally, social isolation and feelings of loneliness impact morbidity and mortality [32]. Isolated people appear to be more at risk of premature death and a shorter healthy life expectancy than non-isolated individuals [7]. A meta-analysis suggested that people with good social connections have a 50% greater chance of survival than those with poor social connections [55].

The main limitation of this study is the fact that 70.4% of the participants were recruited through associations, suggesting that the study population is composed of the most vigorous OAs. Thus, some of our study results may not be extrapolated to the general older population. However, our study may become the basis for the design of more in-depth studies in the future. In addition, the view of GPs on the social health of OAs was not discussed, even though GPs have a central role in the medical follow-up of OAs. The second “Petits Frères des Pauvres” study concluded that the mental health impact of the COVID-19 pandemic was rarely discussed during GP consultations because only 10% of people who consulted after lockdown mentioned the subject [23]. It, therefore, seems important to raise awareness of this potential issue among GPs because the preservation of the social health of patients, especially elderly patients, is an important factor in overall health. Identifying isolated OAs would make it possible to implement adapted solutions in accordance with their needs. Finally, at first sight, the modest size of the sample could be a limitation and thus limit the significance of the results. However, unlike quantitative studies, the samples in qualitative studies do not have to be very large to ensure the validity of the results. In a qualitative study, the number of participants can be stopped as soon as the data saturation effect has been reached; that is how we proceeded [16].

## 5. Conclusions

Most of our elderly population saw DTs as an additional tool for maintaining links with their relatives. While the benefits of DTs are undeniable according to the participants, they also point out confidentiality, privacy, and security risks, and even the danger that the internet can represent. Most participants were equipped with DTs and had been for some time, and the interface was not seen as difficult to use. However, learning to use DTs was tedious for some, particularly for those who had not started using these tools when they were still working. It seems relevant to extend the availability of workshops for DT and ensure that OAs know that the training is available. Individual digital workshops, which were much appreciated by our participants, could be favored. Senior education programs can also be an interesting alternative for intellectual, social, and physical stimulation. However, they do not exist throughout France, especially in rural areas.

Finally, our study may be a basis for the design of future in-depth studies in the general elderly population and not on a particular profile, as seen here because of the method of recruitment suggesting that the study population was composed of the most vigorous OAs.

## Figures and Tables

**Table 1:** Interview guide.

Item	Questions								
Description of Entourage	Family	□ Yes □ No	Friends	□ Yes □ No	Neighbors	□ Yes □ No			
Means of maintaining social links before the 1st lockdown *	Physical link	□ Yes □ No	Virtual link by DTs	□ Yes □ No					
Use of DTs before the 1st lockdown *	Do you have DTs?	□ Yes □ No	Do you know how to use them?	□ Yes □ No	Do you use DTs?	□ Yes □ No	What do you think about the use of DTs by OAs?	Are OAs sufficiently helped by society for Internet use?	□ Yes □ No
	What material do you have?	□ mobile □ Tablet □ Computer	For what purpose(s)?		For what reasons?				
	How did you get it?		At what frequency?						
Collection of experiences during lockdowns	How did you experience the lockdowns in 2020?		Have lockdowns increased your FL?	□ Yes □ No	How did you stay in contact with your social circle?				
Have the lockdowns changed your use of DTs?	Have lockdowns changed your view of DTs?	□ Yes □ No	Have lockdowns changed your need for DTs?	□ Yes □ No	Can DTs reduce social isolation and FL of OAs?	□ Yes □ No			
Opinions on training workshops for DTs	Are you aware of the existence of digital workshops?	□ Yes □ No	Have you already participated ?	□ Yes □ No					
			If yes						
			Group workshops	□ Yes □ No					
			Individual workshops	□ Yes □ No					
			Frequency						
			Were you satisfied?	□ Yes □ No					
			Did you learn new things?	□ Yes □ No					
			Did the training meet your expectations?	□ Yes □ No					
			Describe the practical aspects						
			Is it important to propose these trainings to OAs?	□ Yes □ No					

DT: digital tool, OA: older adult, FL: Feeling of loneliness. * March 2020.

**Table 2:** Social density score developed by the Centre de Recherche pour l’Étude et l’Observation des Conditions de vie, varying from 0 to 8 [17].

Question	Response	Points	Response	Points	Response	Points	Subscore/2
Number of people in the household, including the participant	1	0	2	1	≥3	2	
Does the participant regularly meet close family members?	No	0	Yes	2			
Does the participant receive friends or relatives at his/her home?	Never or rarely	0	Once a month	1	Once a week	2	
Does the participant participate in an association or club?	No	0	Yes, one	1	Yes, several	2	
TOTAL SCORE/8							

**Table 3:** The main characteristics of the participants.

Variable	Age	Sex	Marital Status	Profession before Retirement	NC	NC Living Near	Lodging	Areas of Residence	Number of DTs	Internet Connection	FL	SDS
Participants	80	F	Married	Farmer	3	0	House	Rural	3	Yes	No	7
	80	M	Married	General practitioner	3	0	Apart	Urban	3	Yes	No	6
	80	F	Single	Psychotherapist	0	0	Apart	Urban	3	Yes	No	2
	81	F	Married	Headmaster	2	0	House	Urban	2	Yes	Yes	3
	82	F	Married	School teacher	2	1	House	Rural	3	Yes	No	3
	82	F	Divorced	Executive secretary	0	0	House	Rural	2	Yes	No	4
	82	M	Married	Train driver	3	2	House	Rural	3	Yes	No	3
	82	F	Married	Farmer	3	1	House	Rural	1	No	No	7
	82	F	Married	Relationship manager	2	0	House	Urban	3	Yes	No	5
	82	F	Widow	Bank clerk	4	4	House	Rural	2	Yes	No	6
	83	M	Married	Foreman	2	1	House	Rural	3	Yes	No	4
	84	F	Widow	Housewife	0	0	Apart	Urban	1	No	Yes	2
	84	M	Married	School teacher	1	0	House	Rural	3	Yes	No	7
	84	M	Divorced	Electrician	3	0	House	Rural	2	Yes	No	4
	85	F	Divorced	Nurse	3	2	House	Urban	3	Yes	No	4
	85	F	Widow	Butcher	2	1	House	Rural	2	Yes	No	6
	86	F	Widow	Physicist	2	1	Apart	Urban	3	Yes	No	4
	87	F	Widow	Laboratory technician	4	1	House	Urban	1	No	Yes	5
	87	M	Widower	Mechanic	2	1	Apart	Urban	2	Yes	No	3
	89	F	Widow	Bookkeeping help	2	0	House	Rural	1	No	No	2
	89	F	Widow	Seamstress	3	2	Apart	Urban	1	No	No	5
	89	M	Married	Photographer	3	1	Apart	Rural	2	Yes	No	5
	91	F	Widow	Farmer	5	4	House	Rural	1	No	No	6
	93	M	Married	Farmer	3	1	House	Rural	1	No	No	6
	94	M	Married	Store manager	2	1	House	Rural	2	No	No	7
	98	F	Divorced	Florist	0	0	House	Urban	1	No	No	3
	99	M	Widower	Team manager	1	0	House	Urban	1	No	No	3
Mean ± SD	85.9 ± 5.3				2.2 ± 1.3	0.9 ± 1.1			2 ± 0.8			4.5 ± 1.6

NC: number of children, DT: digital tool, FL: feeling of loneliness, SDS: social density score, F: female, M: male, Apart: Apartment, SD: standard deviation.

## Data Availability

The data presented in this study are available on request from the corresponding author. The data are not publicly available.
